# Hepatoprotective Activity of *Elephantopus scaber* on Alcohol-Induced Liver Damage in Mice

**DOI:** 10.1155/2012/417953

**Published:** 2012-08-29

**Authors:** Wan Yong Ho, Swee Keong Yeap, Chai Ling Ho, Raha Abdul Rahim, Noorjahan Banu Alitheen

**Affiliations:** ^1^Department of Cell and Molecular Biology, Faculty of Biotechnology and Biomolecular Sciences, Universiti Putra Malaysia, Selangor, 43400 Serdang, Malaysia; ^2^Institute of Biosciences, Universiti Putra Malaysia, Selangor, 43400 Serdang, Malaysia

## Abstract

*Elephantopus scaber* has been traditionally used as liver tonic. However, the protective effect of *E. scaber* on ethanol-induced liver damage is still unclear. In this study, we have compared the *in vivo* hepatoprotective effect of *E. scaber* with *Phyllanthus niruri* on the ethanol-induced liver damage in mice. The total phenolic and total flavanoid content of *E. scaber* ethanol extract were determined in this study. Accelerating serum biochemical profiles (including AST, ALT, ALP, triglyceride, and total bilirubin) associated with fat drop and necrotic body in the liver section were observed in the mice treated with ethanol. Low concentration of *E. scaber* was able to reduce serum biochemical profiles and the fat accumulation in the liver. Furthermore, high concentration of *E. scaber* and positive control *P. niruri* were able to revert the liver damage, which is comparable to the normal control. Added to this, *E. scaber* did not possess any oral acute toxicity on mice. These results suggest the potential effect of this extract as a hepatoprotective agent towards-ethanol induced liver damage without any oral acute toxicity effect. These activities might be contributed, or at least in part, by its high total phenolic and flavonoid contents.

## 1. Introduction

Reactive oxygen species (ROS) are continuously generated during metabolic processes to regulate a number of physiological functions essential to the body [1]. ROS are prone to withdraw electron from biological macromolecules such as proteins, lipids, nucleic acids in order to gain stability in the biological system. When the production of ROS exceeds the capability of the body to detoxify these reactive intermediates, oxidative stress would be generated [2]. This may lead to drastic harm to the body such as membrane damage, mutations due to attenuation of DNA molecules, and disruption to various enzymatic activities in metabolism of the body [3–5]. 

Alcohol, a natural product that has been available for human consumption for thousands of years, is a common cause for ROS insult in the liver [6]. Despite the claim that small amount of alcohol consumption may be beneficial for preventing and reducing the mortality rate of coronary heart diseases and ischemic stroke, it should also be noted that alcohol is toxic to almost every organ of the body [7]. Metabolism of alcohol in liver generates excessive free radicals and increased peroxisomal oxidation of fatty acid, which would ultimately affect functionality of the antioxidant systems to eliminate ROS in the body [6]. Therefore, the mechanism to restore hepatic injuries caused by alcoholic oxidative stress is tightly regulated by the antioxidant status of a living system. 

Many plants that portrayed good antioxidant activity are also associated with hepatoprotection potential. Some good examples include *Myristica malabarica *L. [8], *Calotropis gigantea *[9], *Acanthus ilicifolius *[10], *Momordica dioica *[11], and* Phyllanthus niruri *[12]. Members from the Elephantopus family, including *Elephantopus scaber *Linn., *Elephantopus mollis *Kunth., and *Elephantopus tomentosus* have also been shown to possess hepatoprotective activities in rats [13–15]. Additionally, the use of *E. scaber *liver protective purposes was also observed in folk medicinal practices. Back in Brazil, root juice of *E. scaber *had already been consumed for years to heal liver troubles as well as hepatitis [16]. In China, a traditional herbal drink, which is made up of a few herbal products including *E. scaber, *had been claimed to provide protection of the liver against cancer, hemangioma, fatty accumulation, and cirrhosis as well as for anti-hepatitis B [17]. This formulation had also been named as “Yi-Gan-Yin,” which means “drink that is beneficial to the liver” to relate to its function. 

In Taiwan, a folk medicinal formulation (Teng-Khia-U) consisting of *E. scaber, Elephantopus mollis, *and *Pseudelephantopus spicatus, *which was originally devised for treating nephritis, edema, dampness, chest pain, pneumonia, and scabies, was shown to possess hepatoprotective activity against *β*-D-galactosamine-(D-Ga1N-) and acetaminophen-(APAP-) induced acute hepatic damages in rats [14]. *E. scaber *alone could also inhibit carbon-tetrachloride-(CCl4-)induced liver injury [18] and more recently, the plant was reported to show liver protection against lipopolysaccharide-induced liver injury in Sprague-Dawley Rats [19]. Various factors could be responsible for the liver protective ability of this plant. The antioxidant potential of *E. scaber*, which may be attributed to the phenolic and flavonoid content of the plant, may be among the underlying constituents that contributed to this bioactivity. 

Here, we report for the first time evaluation on the hepatoprotection effect of the ethanolic leaf extract of *E. scaber* on ethanol-induced liver injury in mice models. In addition, the total phenolic content and total flavonoid content of the extract were also evaluated in this study. Likewise, the safety of consuming the herb was also a great concern since extracts or decoction of this plant has been applied widely. In this study, we had made an attempt to investigate the oral acute toxicity of this plant extract in mice. This would be crucial to justify the safety of consuming this plant despite its potential to protect the liver against toxic insults. 

## 2. Materials and Methods

### 2.1. Preparation of *E. scaber* Ethanol Extract

The matured leaves of *E. scaber *were collected between 10.00 AM and 11.30 AM of October 18, 2009. The plant was identified and deposited with voucher specimen number FRI65693 in the Forest Research Institute Malaysia (FRIM), Kepong, Selangor. Ethanolic leaf extract of *E. scaber *was prepared as described previously [20]. Briefly, the leaves of *E. scaber* were powdered and extracted using absolute ethanol at room temperature. Extraction was repeated three times and the content of each extraction was mixed and filtered through grade 1 Whatman filter paper. The filtrate was then evaporated to dry under reduced pressure at <40°C using Aspirator A-3S (EYELA, Japan). We obtained a residue of about 8% yield of the initial dried leaves' weight, and this extract was stored at −20°C until use. 

### 2.2. Determination of Total Phenolic Content (TPC)

TPC of the ethanol extract of *E. scaber* was determined according to the method described by Singleton and Rossi (1965) with slight modification. Test samples were first prepared by dissolving the extract into methanol to yield a concentration of 500 *μ*g/mL. Then, 100 *μ*L of each of the sample was added with 500 *μ*L of Folin and Ciocalteu'sphenolreagent and 7.9 mL of distilled water. After 3 minutes, 1.5 mL of Na_2_CO_3_ (20% w/v) was added and the mixture was allowed to stand for 2 hours in dark with intermittent shaking. Absorbance reading was then taken at 765 nm using the *μ* Quant ELISA Reader (Bio-tek Instruments, USA). The measurement was carried out in triplicates and results were expressed as mg ofgallic acidequivalents per g of extract (mg GAE/g of extract).

### 2.3. Determination of Total Flavonoid Content (TFC). TFC of **E*.* scaber*.*

Ethanol extract was determined using the aluminium chloride colorimetric assay [21] with slight modification. First, 250 *μ*L of test sample at concentration of 500 *μ*g/mL was mixed with 1 mL of distilled water and 75 *μ*L of NaNO_2_ (5% w/v). The mixture was let to stand for 5 minutes and added with 75 *μ*L of AlCl_3 _(10% w/v) subsequently. At sixth minute, the solution was added with 500 *μ*L of NaOH (1 M) and the total volume was made up to 2.5 mL with distilled water. The solution was mixed well, and absorbance was measured against the sample's blank at 510 nm. The measurement was carried out in triplicates, and TFC was expressed as mg ofcatechin equivalents per g of extract (mg CE/g of extract). 

### 2.4. Development of Liver Injury and Treatment Administration in Mice Model

Male ICR mice (8 weeks old) with body weight of 25 ± 3 g were used in this experiment. All animals were housedin prebedded plastic cages under controlled conditions of 22 ± 3°C, 55 ± 5% humidity and standard 12 hours of day/dark light cycles. The animals were provided access to standard pellets and tap water *ad libitum* and acclimatized for 2 weeks before starting the experiment. This work has been approved by Animal Care and Use Committee, Universiti Putra Malaysia (UPM), (Ref: UPM/FPV/PS/3.2.1.551/AUP-R2). Then, the mice were randomly divided into 6 groups with 8 mice each, and body weight of each of the mice was measured and recorded. Liver cell damage was induced in 5 groups of mice by feeding with 100 *μ*L of ethanol (50% v/v) p.o. using intragastric tube for 7 consecutive days, while 1 group was fed with 100 *μ*L of phosphate buffer saline to serve as the un-induced control. After 24 hours of the last ethanol dosing, treatment was given to all the mice through oral administration. Grouping and the treatments given to the mice is shown in Table 1. After receiving daily treatment for a total of 7 days, the mice were anesthetized with 2% isoflurane (Merck) and sacrificed by cervical dislocation at 24 hours after the last feed. Blood samples were collected immediately and stored in heparin-coated capillary tubes for analysis of liver enzyme profiling and biochemical analysis. Livers were excised from the animals, washed with normal saline, and then fixed in 10% buffered neutral formalin for histopathological examinations.

### 2.5. Biochemical Analysis

Blood samples collected from the animals were centrifuged at 3000 rpm for 15 minutes, and plasma from each sample was collected for analysis for various biochemical parameters including alkaline phosphatase (ALP), aspartate aminotransferase (AST), alanine aminotransferase (ALT), total billirubin (TB), total cholesterol, (TC) and triglyceride (TG). All the analyses were performed on the Hitachi 902 Automatic Analyzer using the adapted reagents from Roche (Germany).

### 2.6. Histopathological Evaluation

Tissues were dehydrated in ascending grades of ethanol (70% to 100%) and cleared in xylene with the use of TP1020 automated tissue processor (Leica, Germany) after fixing in 10% formalin for overnight. Then, the tissues were embedded in paraffin wax blocks on the EG 1140 H embedding station (Leica, Germany), section to 4 *μ*m thick using Jung Multicut 2045 microtome (Leica, Germany) and mounted on glass slides. After that, deparaffination was carried out by immersing the slides in Xylol for 3 minutes and then in 100% ethanol for 1 minute, 96% ethanol for 1 minute, 80% ethanol for 1 minute, and 60% ethanol for 1 minute. Lastly, the slides were stained with haematoxylin (Sigma, USA) and eosin (Sigma, USA) and then mounted under coverslips using DPX mounting medium (BDH Laboratory, England). Histological changes in the tissue sections were examined and captured under the BX51 light microscope (Olympus, Japan).

### 2.7. Study on Acute Oral Toxicity in Mice

Acute Oral toxicity of *E. scaber* ethanol extract was performed according to the OECD guidelines 425 using female Balb/C mice aging 8–10 weeks old. All animals were housedin prebedded plastic cages under controlled conditions of 22 ± 3°C and standard 12 hours of day/dark light cycles. The animals were provided access to standard pellets and tap water *ad libitum* and acclimatized for 2 weeks before starting the experiment. The mice were fasted 4 hours prior to the experiment and then divided into groups of five. Body weight of each of the mice was measured and recorded after fasting. Then, animals in the treatment group were fed with single oral dose of *E. scaber* ethanol extract (5000 mg/kg BW) that was dissolved in PBS, while the control group received PBS solution only (10 mL/kg BW). The mortality, body weight, toxic symptom, and behavior of the animals were observed carefully at 15 and 30 minutes, 1, 2, 4, 12, 18, and 24 hours after oral administration and daily thereafter over a total period of 14 days. 

### 2.8. Statistical Analysis

Results were expressed as mean ± standard error (SEM). Single comparison for differences between means was determined by Student's *t*-test, while multiple comparisons were evaluated using ANOVA test followed by Duncan test. A level of *P* ≤ 0.05 was taken as statistically significant.

## 3. Results

### 3.1. Total Phenolic and Total Flavonoid Content in *E. scaber*

The presence of high flavonoids and phenolic compounds in plants had been identified to contribute to their antioxidant values [22]. Our results indicated that the total phenolic content of *E. scaber *was 193.05 ± 1.17 mg GAE/g of the extract. while the total flavonoid content was 120.87 ± 0.61 mg CE/g of extract. 

### 3.2. Hepatoprotective Effect of *E. scaber* on Ethanol-Induced Liver Damage in Mice

Ethanol treatment resulted in a significant elevation of serum ALT, ALP, and AST levels in comparison to the control group (Table 2). However, the amount of these enzymes reduced after treatment by various concentrations of *E. scaber *as well as by *P*. *niruri* in general. The decrease of serum ALT concentration after treatment with ESH was greatest among the three concentrations of the extract, but ESL and ESM were capable of restoring the amount of ALT to near normal value. Overall, the reduced ALT level after treatment by all concentrations of *E. scaber *showed no significant difference from the normal control, while the level was significantly (*P* < 0.05) lower than the control group after treatment by *P*. *niruri*. Similarly, the level of serum ALP was also restored to normal by most of the treatments although differences between all groups were not statistically significant. Only treatment by ESL caused a slight increase in the ALP level, which was not significantly different from the normal control group. Treatment with ESH resulted in a reduction to ALP level that was closest to the normal control group. On the other hand, the effect of ethanol on AST level in mice was much more apparent than the other two liver enzyme markers. A marked increase (2.5 fold) was observed upon induction by ethanol. In spite of this, treatment with either *E. scaber* or *P*. *niruri* successfully reduced the AST concentrations in mice to a level that were not statistically different from the control group. Comparing between the 3 concentrations of *E. scaber*, the level of AST was most significantly reduced by ESH and the value was nearest to the normal control. Reduction by treatment with *P*. *niruri* was greater than reduction by all concentrations of *E. scaber* but the AST level was even lower than the normal control group.

On the other hand, differences in the triglyceride (TG) and total bilirubin (TB) content between the control and the treatment groups were not statistically significant. Seven days of preinduction with ethanol resulted in a slight increase of TG and TB content as compared to the normal control group (Table 2). Following treatment for 7 consecutive days by *E. scaber *or *P. niruri* extracts, both serum TG and TB content in the mice decreased. ESH resulted in the greatest reduction of both TG and TB concentration. However, the final TG level after treatment by ESM and the final TB level after treatment by ESL were found to be closest to the normal control group. 

### 3.3. Effect of *E. scaber* on Alcohol-Induced Lesion in Mice Liver

**Figure 1:**
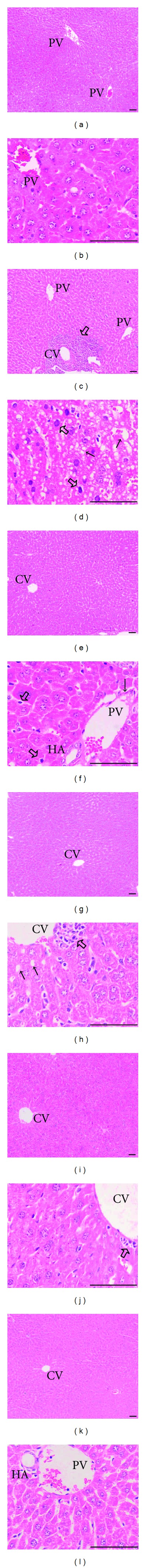
Histological micrograph of liver sections of mice stained with haematoxy lin and eosin. Liver sections excised from the normal control mice (a, ×40) showed normal structure of the hepatocytes with defined cell lining and round nuclei (b, ×100). Histological sections of mice fed with 50% ethanol showed damage of the liver structure as indicated by infiltration of monocytes (block arrow head) (c, ×40), steatosis (filled arrow), and the presence of necrotic cells (block arrow) (d, ×100). Treatment with 15 mg/kg BW of *P. niruri*, the positive control, indicated normalization of the damaged hepatocytes (e, ×40). A great reduction of fat droplets (filled arrow), and the presence of a minute quantity of necrotic cells (block arrow) (f, ×100) could also be observed in this group. On the other hand, section of liver treated by ESL showed recovery of hepatocytes into normal shape (g, ×40), along with lesser fat droplets (filled arrow) and mild monocytes infiltration (block arrow) (H, ×100). When the concentration was increased to 15 mg/kg BW (i, ×40), mild monocytes infiltration was observed (j, ×100). Upon treatment with ESH, structure of the hepatocytes reverted to normal (k, ×40) with defined cell lining and round nuclei (l, ×100). Centrilobular vein (CV), peripheral vein (PV), hepatic artery (HA). Bar = 50 *μ*m.

Liver sections excised from mice from the control group, which did not receive pretreatment with alcohol, showed normal structure of the hepatocytes that were polyhedral in shape (Figure 1(a)). The cells exhibited well-preserved cell lining with well-defined cytoplasm and nucleus (Figure 1(b)). In contrast, pretreatment of 50% ethanol resulted in damage to the liver. Infiltration of mononuclear cells was detected in the liver section (Figure 1(c)), and normal structure of the hepatocytes was found to be distorted where the cells were rendered with undefined lining and loss of nucleus (Figure 1(d)). Besides, the presence of small foci of necrotic cells and the occurrence of steatosis or accumulation of fatty droplets was detected in many regions of the liver sections.

After treatment by *P. niruri* for 7 days, liver damage of the mice was recovered, as shown in Figure 1(e). When viewed under a higher magnification (Figure 1(f)), it was noted that recovery of the cells had not been completed where a small number of necrotic cells as well as very few lipid droplets were still observable in the liver sections. However, the abnormalities that were detected in the ethanol control group reduced significantly after treatment. Similarly, the administration of ESL after the alcohol induction also showed that regeneration of the hepatocytes had taken place but the lining of the cells had not been recovered completely (Figure 1(h)). The presence of mild monocytes infiltration and a few fat droplets were still detectable in the liver section. Likewise, normalization of hepatocytes into normal structure also occurred when the concentration of *E. scaber* was elevated. However, the recovery was more significant in these two groups where undefined lining of the cells were reverted to normal and distinct nucleus could be seen (Figures 1(i) and 1(k)). In addition, the group treated by ESM showed a greater reduction of monocyte infiltrate), and the lipid droplets were absent in comparison to the low dosage group (Figure 1(j)). The mice treated by ESH showed normal morphology of the hepatocytes, along with normal appearance of the centrilobular vein, peripheral vein and hepatic artery (Figure 1(l)), resembling the tissue architecture of liver section from the untreated control group. 

### 3.4. Oral Acute Toxicology Study in Mice

Acute oral toxicity is defined as the adverse effects that can be observed after oral administration of a test substance at either single dose or multiple doses, which is given within 24 hours [23]. Therefore, median lethal oral dose (LD_50_), which is defined as the statistically derived single dose of a substance that can kill 50% of animals when administered orally, can be determined form this assay. In this study, the acute oral toxicity assay was carried out following OECD guideline no. 423 (2011). No mortality was observed after 14 days of treatment with a limit dose of 5000 mg/kg BW of *E. scaber*. All the treated mice could tolerate with the extract given with no signs of abnormalities or gross lesions in necropsy findings (Table 3). No statistically significant differences in body weight and weight gain were noted between the treated group and the untreated control group (Table 4). Thus, LD_50_ of *E. scaber* could not be determined from this study and the extract could be regarded as nontoxic for oral consumption up to a concentration of 5000 mg/kg BW in mice.

## 4. Discussion

The liver plays a pivotal role in the biological system that is responsible for the metabolism and clearance of drugs and xenobiotics, including ROS [24]. Liver has become the central organ for detoxification as the liver cells (hepatocytes), the main components that make up the organ, contain majority of enzymes that are responsible for drug metabolism of the entire body [25]. However, when the amount of drugs or xenobiotics that is encountered has exceed the maximum metabolic capability of the liver; damaging effect of the toxins may lead to various liver ailments. Overconsumption of alcohol had been associated to a spectrum of liver injuries with varying degree of severity, with some common pathologies including steatosis, foamy degeneration, steatonecrosis, venous lesion, and cirrhosis [26].

Generally, alcohol is metabolized in the liver as a process of detoxification. The metabolism of alcohol occurs mainly via alcohol dehydrogenases (ADH), which requires the cofactor NAD^+^. The reduced form of NAD^+^ (NADH) is attenuated when the alcohol concentration is in excess, and this could cause hepatic NADH accumulation [7]. As a result, more fatty acids and triglycerides would be synthesized whereas *β*-oxidation of fatty acids will be impeded [7]. Accumulation of ROS and polyunsaturated fatty acids would increase the oxidative stress and toxicity to the hepatic cells [27]. In this study, the increased level of ALT, ALP and AST, as well as elevated TG and TB content (Table 2) after 7 days of continual feeding with high concentration (50% v/v) of ethanol were indications for alcohol intoxication to the liver. Besides, results from histological images that showed accumulations of fatty droplets in the hepatocytes also provided clear evidence that the preinduction with 50% of ethanol induced liver damage, including loss of cell membrane integrity, accumulation of fatty acids, and necrotic cell death in the mice (Figures 1(c) and 1(d)). Treatment with ESL, ESM, and ESH was able to reduce the accumulation of fats in the mice (Figures 1(e), 1(f), 1(g), 1(h), 1(i), and 1(j)) and decline of TG content in the serum (Table 2). Moreover, the group treated by ESH showed the most substantial improvement of steatosis and the greatest reduction of TG concentration. The ethanolic extract of *E. scaber *was chosen for the present study as it was shown to exhibit a wide range of bioactivities and most of the compounds in *E. scaber *were isolated from this extract [28]. Therefore, it was hypothesized that the *E. scaber *ethanol extract would show potent liver protection activity against ethanol-induced liver damage. 

In contrast, the recovery of liver injury by treatment with *P. niruri* was less significant. When comparing histological appearance of the hepatic cells and TG content to ESH treatment group, mild monocytes infiltration and a few fat droplets were still detectable in the former group. On the other hand, increase of ALP and TB concentrations in the serum of alcohol-induced mice might reflect blockage of the bile ducts that obstructed the secretion of bile. Hepatic ALP is present on the lining of biliary ducts and is secreted via biliary bile into blood circulation [29]. ALP synthesis is stimulated during pathological conditions like bile duct obstruction, primary biliary cirrhosis, primary sclerosing cholangitis, drug-induced cholestasis, adult bile ductopenia, metastatic liver diseases, and bone diseases [30]. Bilirubin, on the other hand, is a bile pigment produced from the enzymatic breakdown of heme within the reticuloendothelial system where its elevation in bloodstream could be related to increased bilirubin production (or increased hemolysis), decreased conjugation, or defects in bilirubin transport [31]. Therefore, both ALP and TB levels measure how well the liver functioned instead of the extent of hepatic injury. Treatment by ESH was most effective in reducing the concentrations of ALP and TB that were elevated by alcohol in comparison to treatment by ESL, ESM, and *P. niruri*. Therefore, it could be deduced that *E*.* scaber *provided protection to the liver against harmful effect of alcohol by preserving the functionality of this organ. 

In this study, *P. niruri* was used as the positive control as this plant had been studied extensively and was shown to possess protective activity against liver injuries that were caused by a number of hepatotoxins including acetaminophen [32], nimesulide [33], carbon-tetrachloride [34–36], and paracetamol [37]. Biochemical analysis from this study was in good agreement with previous finding where *P. niruri* was capable of restoring levels of the liver enzymes alanine transaminase (ALT) and aspartate transaminase (AST) that were elevated following liver injury induced by alcohol [32] (Table 2). ALT is found primarily in the cytoplasm of hepatic cells, while AST is present in both cytoplasm and mitochondria [29]. Both the enzymes catalyse gluconeogenesis from noncarbohydrate sources and are important markers for liver injury [30]. Elevation of the serum concentrations of these 2 markers implied disruption of plasma membrane integrity, which eventually lead to leakage of the enzymes into the blood circulation [31]. Similar to the positive control group, treatment with *E. scaber *also successfully decreased the elevated concentrations of AST and ALT in a dosage-dependent manner whereby the highest concentration of *E. scaber *(ESH) reduced both ALT and AST most. Restoration of both AST and ALT levels suggested that *E. scaber *could potentially restore hepatic cells, or more specifically the integrity of cell membrane and the normalization of hepatocytes into their normal tissue architecture. 

The reduced concentrations of both these aminotransferases were consistent with previous studies on lipopolysaccharide-(LPS-) treated rats, which showed that theprotective effect of *E. scaber *over LPS-stressed acute hepatic injury was contributed in part by the antioxidant property of the herb [19]. Previously, the leaf, stem, and root of *E. scaber *had been shown to exhibit antioxidant activities [38, 39] and this property had also been suggested to be attributed by its constituting phenolic content [38]. A few studies have also shown that the hepatoprotective activity is highly correlated to the phenolic content of a given plant and the flavonoid content was suggested as the main contributor to this bioactivity [40–42]. Therefore, the hepatoprotective activity against ethanol-induced liver damage that was demonstrated by the *E. scaber* ethanolic extract could be attributed to the high phenolic and flavonoid content of the plant. However, this would require further assessment through identification and screening of the phenolic constituents of this plant extract. 

From this study, ethanol extract of *E. scaber *leaves showed promising hepatoprotection activity in mice with alcohol-induced liver damage. The ability of *E. scaber*-treated mice to cope with the oxidative stress induced by alcohol could be accountable to the antioxidant capacity of the herb. These activities might be contributed, or at least in part by its high total phenolic and flavonoid contents. On the other hand, it was also noted that the hepatoprotective effect of the extract was concentration dependent. Whilst 30 mg/kg BW appeared to most effectively restore the liver damage to near normal, lower concentrations of the extract also showed moderate liver protection activities. More importantly, all the concentrations of *E. scaber* selected for this study were lower than the highest concentration used in acute oral toxicology study (5000 mg/kg BW). With validation from the safety of consuming the ethanol extract of *E. scaber, *this plant extract may be useful as a natural protecting agent against liver damage.

## Figures and Tables

**Table 1:** Induction and treatment given to 6 groups of mice for liver protection study.

Group	Preinduction with 50% (v/v) ethanol	Treatment through oral administration (7 days)	Amount of treatment given
Control	None	Phosphate buffer saline	1 x
Ethanol + PBS	7 days	Phosphate buffer saline	1 x
Ethanol + ESL	7 days	*E. scaber *ethanol extract	3 mg/kg BW
Ethanol + ESM	7 days	*E. scaber *ethanol extract	15 mg/kg BW
Ethanol + ESH	7 days	*E. scaber *ethanol extract	30 mg/kg BW
Ethanol + PN	7 days	*Phyllanthus niruri*	15 mg/kg BW

**Table 2:** Effect of *E. scaber* on activities of serum marker enzymes, triglyceride, and total bilirubin content in ethanol-induced mice.

Group	ALT (U/L)	ALP (U/L)	AST (U/L)	TG (mmol/L)	TB (mmol/L)
Control	81.9 ± 17.8^#^	84.0 ± 10.9^#^	187.4 ± 26.3^#^	1.07 ± 0.35	177.6 ± 21.2
Ethanol + PBS	172.2 ± 2.9*	118.0 ± 3.0*	902.8 ± 16.7*	1.53 ± 0.45	228.0 ± 19.2
Ethanol + ESL	83.8 ± 2.2*	119.0 ± 11.0^#^	434.8 ± 55.6*	1.54 ± 0.38	198.4 ± 40.9
Ethanol + ESM	82.8 ± 18.9^#^	99.7 ± 17.0	389.4 ± 14.6^#^	1.20 ± 0.19	189.7 ± 45.3
Ethanol + ESH	69.7 ± 5.6^#^	90.7 ± 13.3	348.2 ± 6.3^#^	0.70 ± 0.09^#^	136.3 ± 42.8
Ethanol + PN	48.55 ± 2.9^∗,#^	146.0 ± 3.0*	229.8 ± 16.7^#^	1.12 ± 0.45	180.9 ± 19.2

Value represents mean values ± SEM of 8 mice. *Statistical significance (*P* < 0.05) compared to mice in the untreated control group, ^#^Statistical significance (*P* < 0.05) compared to mice in ethanol-induced group.

**Table 3:** Clinical signs and necropsy findings in mice after acute treatment with *E. scaber*.

Treatment group	No. of mice	Clinical sign	Gross necropsy finding	Mortality
Untreated control	8	No observable abnormalities	No findings	None
*E. scaber*	8	No observable abnormalities	No findings	None

**Table 4:** Effect of acute treatment with *E. scaber* ethanol extract on the weight of mice.

		Mean average values (g)	
		Untreated control	*E. scaber*
Body weight	before	20.40 ± 1.18	21.95 ± 0.53
	after	23.09 ± 1.97	23.45 ± 0.65
Body weight gain		2.69 ± 1.19	1.50 ± 0.90

Data represents mean ± SEM of sample size (*n*) of 8. *Statistical significance (*P* < 0.05) between the control cells and treatment groups.
